# 

**Published:** 1818-02

**Authors:** 


### CORRESPONDENCE.

The Communication referred to in Dr. Forbes's last letter came safely to hand, and is under consideration.

---

☞ Authors and Publishers are requested to send Copies of new Works for Review as early as possible after their publication. They will be reviewed early in proportion.

